# Séroprévalence et facteurs associés à *Toxoplasma gondii* chez les femmes enceintes vues en consultation prénatale à Douala, Cameroun: une étude transversale

**DOI:** 10.11604/pamj.2026.53.103.50400

**Published:** 2026-02-26

**Authors:** Bernardin Jeff Tsimi Mala, Djibrila Yaouba, Kelly Ambassa Edoa, Amanda Billy Deba Adidigue, Charles Kevy Tsimi Messomo

**Affiliations:** 1 Faculté des Sciences de la Santé et Médicales, Université Internationale Kesmonds, Ngaoundéré, Cameroun,; 2 Délégation Régionale de la Santé Publique de l'Adamaoua, Ngaoundéré, Cameroun,; 3 Faculté des Sciences, Université de Ngaoundéré, Ngaoundéré, Cameroun,; 4 Hôpital Régional de Ngaoundéré, Ngaoundéré, Cameroun,; 5 Institut Supérieur des Sciences, de Technologie, de Management et du Développement Durable, Ngaoundéré, Cameroun,; 6 Ecole de Formation des Personnels de Santé Fondation Bobuin, Douala, Cameroun,; 7 Faculté de Médecine et des Sciences Biomédicales, Université de Yaoundé I, Yaoundé, Cameroun

**Keywords:** Séroprévalence, facteurs associés, *Toxoplasma gondii*, femmes enceintes, consultation prénatale, Seroprevalence, associated factors, Toxoplasma gondii, pregnant women, antenatal care

## Abstract

**Introduction:**

un diagnostic précoce de la toxoplasmose pendant la grossesse est hautement souhaitable, permettant une intervention rapide en cas d'infection. Cette étude visait à déterminer la séroprévalence de l'infection à T. gondii et ses facteurs associés.

**Méthodes:**

il s'est agi d'une étude transversale menée de novembre 2024 à septembre 2025 dans deux formations sanitaires de la ville de Douala. Nous avons procédé à un échantillonnage consécutif et exhaustif des femmes vues en consultation prénatale à l'Hôpital Gynéco-Obstétrique et Pédiatrique ainsi qu'à l'Hôpital Laquintinie, après obtention de leur consentement éclairé. Les données ont été collectées grâce à un questionnaire, préalablement prétesté. Un modèle de régression logistique binaire multiple a été employé afin d'identifier les facteurs associés, avec un seuil de significativité établi à 5%.

**Résultats:**

cette étude a inclus 305 femmes gestantes, présentant une moyenne d'âge de 29,4 ± 5,9 ans. Plus de 75% des participantes démontraient un manque de connaissances concernant la toxoplasmose. L'étude a révélé une séroprévalence globale des anticorps anti-T. gondii (IgG et/ou IgM) de 74,4% (IC à 95% = 68,9 à 79,3). L'âge (ORa: 1,10, IC 95% 1,03-1,18; p = 0,00); le contact avec les chats (Ora: 0,17, IC 95% 0,06-0,51; p = 0,00); le niveau de connaissances sur la toxoplasmose (ORa: 0,23, IC 95% 0,06-0,81; p = 0,02) et l'habitude de goûter la viande pendant sa cuisson (ORa: 0,45, IC 95% 0,22-0,90; p = 0,02) étaient significativement associés à la séropositivité de la toxoplasmose.

**Conclusion:**

la prévalence générale des anticorps anti-Toxoplasma gondii dans notre population s'est révélée élevée, avec des facteurs d'influence multiples. Il est nécessaire d'établir des programmes de prévention primaire visant à sensibiliser les femmes enceintes aux risques liés à la toxoplasmose durant leur grossesse.

## Abstract (en)

**Introduction:**

early diagnosis of toxoplasmosis during pregnancy is highly desirable, allowing prompt intervention in cases of infection. The purpose of this study was to determine the seroprevalence of Toxoplasma gondii infection and its associated factors.

**Methods:**

we conducted a cross-sectional study from November 2024 to September 2025 in two health facilities in the city of Douala. Consecutive and exhaustive sampling was performed among women attending antenatal care at the Gynaecology-Obstetrics and Paediatrics Hospital and at Laquintinie Hospital after obtaining informed consent. Data were collected using a pretested questionnaire. A multivariable binary logistic regression model was used to identify associated factors, with a significance threshold set at 5%.

**Results:**

this study included a total of 305 pregnant women, with a mean age of 29.45 ± 5.92 years. More than 75% of participants demonstrated poor knowledge regarding toxoplasmosis. The study revealed an overall seroprevalence of anti-T. gondii antibodies (IgG and/or IgM) of 74.4% (95% CI: 68.9 - 79.3). Age, contact with cats, level of knowledge about toxoplasmosis, and the habit of tasting meat during cooking were significantly associated with toxoplasmosis seropositivity.

**Conclusion:**

the overall seroprevalence of anti-T. gondii antibodies observed in this study suggests the need for primary preventive interventions aimed at reducing the prevalence of this infection in the population.

## Introduction

La toxoplasmose est une affection zoonotique largement répandue provoquée par le parasite protozoaire *Toxoplasma gondii* (*T. gondii*) [1]. Il est estimé qu'environ un tiers de la population mondiale a été exposée à *T. gondii* [2]. La contamination se produit principalement par: ingestion d'aliments ou d'eau contaminés par des oocystes excrétés par les chats; consommation de viande insuffisamment cuite ou crue contenant des kystes tissulaires ou congénitalement par transmission transplacentaire de tachyzoïtes [2,3]. Les infections primaires à *T. gondii* contractées pendant la grossesse sont généralement asymptomatiques pour la femme enceinte, mais peuvent entraîner de graves complications néonatales [4]. La toxoplasmose pendant la grossesse a été associée à une fausse couche, une hydrocéphalie, une calcification cérébrale et une choriorétinite chez le nouveau-né [5]. La primo-infection à *T. gondii* au cours du troisième trimestre de la grossesse comporte un risque plus élevé de transmission congénitale que si elle est acquise au cours du premier trimestre.

Dans les pays développés, la toxoplasmose congénitale touche 0,01% à 0,1% des nourrissons [6]. Un diagnostic précoce pendant la grossesse est hautement souhaitable, permettant une intervention rapide en cas d'infection [7]. Le diagnostic de la toxoplasmose peut être établi par la détection directe du parasite ou par des techniques sérologiques, bien que ces dernières présentent des limites dans la détection ou la différenciation des souches parasitaires [3,8]. Bien que des anticorps spécifiques de Toxoplasma (immunoglobulines) soient détectés dans le sérum des femmes enceintes dans les unes à deux semaines suivant l'exposition à l'infection, les résultats sérologiques de ces immunoglobulines sont souvent incapables de différencier les infections aiguës des infections chroniques. La détection d'anticorps immunoglobuline M (IgM) ou d'anticorps IgM et d’immunoglobuline G (IgG) indique une infection aiguë, tandis que des résultats négatifs peuvent suggérer une infection très récente ou l'absence d'infection [1]. La littérature scientifique présente diverses études internationales et africaines mettant en évidence la prévalence et les déterminants de la toxoplasmose. Au Cameroun, plusieurs recherches sur le toxoplasme ont été conduites dans différentes régions, notamment à l'Ouest, au Nord-Ouest et au Centre [9-11]. Dans la région du Littoral, particulièrement à Douala, les publications scientifiques demeurent limitées sur cette thématique, à l'exception de l'étude de Njunda Al *et al*. [7] en 2009, évaluant la séroprévalence des anticorps anti-*Toxoplasma gondii* chez les femmes enceintes de l'Hôpital général de Douala, ainsi que celle menée par Nguefack *et al*. [12] en 2016, justifiant ainsi notre travail actuel. Cette étude visait à déterminer la séroprévalence de l'infection à *T. gondii* et à identifier ses facteurs associés chez les femmes consultant pour le suivi prénatal à l'Hôpital Gynéco-Obstétrique et Pédiatrique de Douala et à l'Hôpital Laquintinie de Douala.

## Méthodes

**Conception et cadre de l'étude:** une étude transversale a été menée afin de déterminer la séroprévalence de l'infection à *T. gondii* et ses facteurs associés à Douala. Cette recherche a été conduite dans deux formations sanitaires (FOSA) de référence de Douala, capitale économique camerounaise: l'Hôpital Gynéco-Obstétrique et Pédiatrique de Douala (HGOPED), établissement de première catégorie localisé dans le District de Santé de Japoma (arrondissement de Douala troisième), et l'Hôpital Laquinitinie de Douala (HLD), FOSA de deuxième catégorie situé dans le District de Santé de Deido (arrondissement de Douala premier), Département du Wouri, Région du Littoral. L'étude s'est déroulée de janvier à septembre 2025, avec une collecte de données sur le terrain effectuée de juin à août 2025.

**Population d'étude:** la population étudiée comprenait les femmes enceintes consultant pour un suivi prénatal à l'HGOPED et à l'HLD, ayant donné leur consentement éclairé. Nous avons appliqué un échantillonnage consécutif et exhaustif. Ont été exclues de l'étude les femmes enceintes consultant dans ces deux établissements qui avaient retiré leur consentement ou choisi librement de quitter l'étude, celles ne disposant pas de résultat de test de toxoplasmose, et celles n'ayant que partiellement complété le questionnaire d'étude. La taille de l'échantillon cible a été déterminée en utilisant une proportion estimée de 82,7% de femmes enceintes infectées par *Toxoplasma gondii*. Cette estimation provenait d'une recherche antérieure évaluant la séroprévalence de la toxoplasmose et ses facteurs de risque de transmission dans trois centres de santé à Dschang, Cameroun [9]. Avec un intervalle de confiance de 95% et une marge d'erreur de 5%, nous avons calculé un échantillon nécessaire d'environ 220 femmes enceintes. Pour anticiper les non-réponses, nous avons appliqué une marge supplémentaire de 5%, portant ainsi la taille minimale finale de l'échantillon à 231 participantes.

**Collecte des données:** la collecte s'est effectuée au moyen d'un questionnaire structuré sur papier, préalablement validé par un test dans chaque FOSA. Une étudiante en fin d'études de sage-femme ayant reçu une formation spécifique a procédé à la collecte après avoir obtenu le consentement éclairé des participantes. L'ensemble des informations a été saisi via le logiciel CS Pro version 7.5 et conservé sur un ordinateur protégé par mot de passe.

**Définitions:** l'étude a recueilli diverses catégories de variables. Les données sociodémographiques comprenaient l'âge, la profession, le niveau d'éducation, le domicile et la situation matrimoniale. Parmi les antécédents obstétricaux documentés figuraient le nombre d'accouchements et la durée gestationnelle. Les facteurs de risque de toxoplasmose identifiés incluaient la vie commune avec des félins, les antécédents transfusionnels et la principale source d'approvisionnement en eau.

**Analyse statistique:** après la collecte, les données étaient examinées et ensuite entrées dans le modèle de saisie de données développé à l'aide du logiciel CS Pro version 7.5. Les données étaient ensuite exportées vers IBM SPSS 23 puis analysées. Les données ont été exprimées sous forme de moyenne ± écart-type pour les variables quantitatives, d'effectifs et de fréquences pour les variables qualitatives. Un modèle de régression binaire multiple a été utilisé afin d'identifier les facteurs associés à la séroprévalence de l'infection à *T. gondii* et le rapport de cotes ajustés pour exprimer la dépendance. Le seuil de significativité a été fixé à 5%.

**Considérations éthiques:** l'étude adhérait aux principes éthiques énoncés dans la déclaration d'Helsinki (2013). Les participantes bénéficiaient d'informations exhaustives, tant écrites qu'orales. Le formulaire de consentement stipulait expressément la nature facultative de leur participation et garantissait l'absence de conséquences négatives en cas de non-participation. Toutes les participantes ont formalisé leur accord par une signature préalable à leur entrevue. Cette étude a reçu l'approbation de l'HLD sous le numéro d'autorisation N° 1474 AR/MINSANTE/DHL/SG ainsi que de l'HGOPED sous le numéro d'autorisation N° 2025/0926/L/HGOPED/DG/DGA/DFRI/CSRI/mae.

## Résultats

**Caractéristiques générales:** cette étude a regroupé 305 femmes enceintes, âgées de 16 à 43 ans, avec un âge moyen de 29,4 ± 5,9 ans. La période gestationnelle s'étendait de 10 à 40 semaines d'aménorrhée (SA), pour une moyenne de 25,8 ± 7,1 SA. Plus de la moitié des participantes avaient poursuivi des études supérieures. Moins de 10% résidaient en milieu rural, et une minorité était mariée, la plupart étant célibataires. Environ 45% des femmes interrogées hébergeaient un chat à leur domicile, tandis que moins de 35% étaient en contact avec ces animaux. Par ailleurs, près de 40% des participantes étaient régulièrement en contact avec la terre, et pourtant moins de 10% utilisaient des gants de protection lors de ces manipulations. Concernant les antécédents médicaux, moins de 30% avaient bénéficié d'une transfusion sanguine. L'approvisionnement en eau des participantes était varié, légèrement plus de la moitié utilisant l'eau de forage, et moins de 5% s'alimentant à partir de puits (Tableau 1).

**Tableau 1:** caractéristiques générales des femmes vues en CPN aux hôpitaux Gynéco-Obstétrique et Pédiatrique de Douala et Laquinitinie de Douala

Variables	Fréquence (N=305)	Pourcentage (%)	IC à 95%
**Niveaux d'instruction**			
Non instruite	1	0,3	0,0 - 1,0
Primaire	12	3,9	2,0 - 6,2
Secondaire	95	31,1	25,9 - 36,4
Universitaire	197	64,6	59,0 -70,2
**Zone de résidence**			
Rurale	25	8,2	5,2 - 11,5
Urbaine	280	91,8	88,5 - 94,8
**Statut matrimonial**			
Célibataire	167	54,8	48,9 - 60,3
Divorcée	1	0,3	0,0 - 1,0
Mariée	137	44,9	39,3 - 50,5
**Age gestationnel**			
Premier trimestre	16	5,2	3,0 - 7,9
Deuxième trimestre	153	50,2	44,6 - 55,7
Troisième trimestre	136	44,6	39,0 - 44,8
**Présence de chat à la maison**			
Oui	130	42,6	36,7 - 48,2
Non	175	57,4	51,8 - 63,3
**En contact avec les chats**			
Oui	101	33,1	27,2 - 38,4
Non	204	66,9	61,6 - 72,8
**Contact avec le sol**			
Oui	117	38,4	32,8 - 43,9
Non	188	61,6	56,1 - 67,2
**Utilisation des gants lors du contact avec le sol**			
Oui	19	6,2	3,6 - 9,2
Non	286	93,8	90,8 - 96,4
**Antécédent de transfusion sanguine**			
Oui	83	27,2	22,3 - 32,1
Non	222	72,8	67,9 - 77,7
**Principale source d'approvisionnement en eau**			
Forage	168	55,1	49,2 - 60,3
Puit	14	4,6	2,6 - 7,2
Minérale	122	40,0	35,1 - 45,2
Autres	1	0,3	0,0 - 1,0

**Antécédents obstétricaux et niveaux de connaissances sur la toxoplasmose:** moins de la moitié des femmes ont signalé des antécédents obstétricaux. Les fausses couches dominaient ces cas (30%), alors que les naissances d'enfants morts représentaient 5%. Toutefois, plus de 50% des femmes avaient accouché plusieurs fois. Notre étude montre que 88,5% des participantes manquaient de connaissances (Tableau 2).

**Tableau 2:** antécédents obstétricaux, parité et niveaux de connaissances des femmes vues en consultation prénatale à l'Hôpital Gynéco-Obstétrique et Pédiatrique de Douala ainsi qu'à l'Hôpital Laquinitinie de Douala

Variables	Fréquence (N=305)	Pourcentage (%)	IC à 95%
**Antécédents obstétricaux**			
Avortement spontané	96	31,5	26,2 - 37,0
Malformation (s)	2	0,7	0,0 - 1,6
Mortinaissance	10	3,3	1,3 - 5,6
Pas d'histoire	197	64,6	58,7 - 69,5
**Parité**			
Primipare	135	44,3	39,0 - 49,8
Multipare	170	55,7	50,2 - 61,0
**Niveaux de connaissances sur la toxoplasmose**			
Absence de connaissances	270	88,5	84,6 - 91,8
Bonnes connaissances	35	11,5	8,2 - 15,4

**Prévalence des anticorps anti-Toxoplasma dans la population étudiée:** l'étude menée auprès de 305 femmes enceintes âgées de 16 à 43 ans, suivies en consultation prénatale à l'Hôpital Gynéco-Obstétrique et Pédiatrique de Douala ainsi qu'à l'Hôpital Laquintinie de Douala, a révélé une séroprévalence globale des anticorps anti-*T. gondii* (IgG et/ou IgM) de 74,4% (IC à 95% = 68,9 à 79,3). La distribution des séroprévalences indique que 72,8% des femmes présentaient des anticorps IgG, 8,5% des anticorps IgM, et 6,6% possédaient simultanément les anticorps IgG et IgM (Tableau 3).

**Tableau 3:** prévalence de l'infection à *Toxoplasma gondii* chez les patientes consultant pour leur suivi prénatal à l'Hôpital Gynéco-Obstétrique et Pédiatrique de Douala ainsi qu'à l'Hôpital Laquinitinie de Douala

Variables	Fréquence (N=305)	Pourcentage (%)	IC à 95%
**Prévalence combinée**			
**IgG et/ou IgM**			
Séropositives	227	74,4	68,9 - 79,3
Séronégatives	78	25,6	20,7 - 31,1
Séronégatives	279	91,5	88,5 - 94,4
Séropositives	26	8,5	5,6 - 11,5
**IgG**			
Séronégatives	83	27,2	22,3 - 32,5
Séropositives	222	72,8	67,5 - 77,7
**IgG et IgM**			
Séronégatives	285	93,4	90,8 - 96,1
Séropositives	20	6,6	3,9 - 9,2

Ig G: immunoglobuline de classe G Ig M: immunoglobuline de classe M

**Facteurs associés à la séropositivité à Toxoplasma:** pour obtenir une analyse plus précise et éliminer les facteurs de confusion, nous avons effectué une régression logistique binaire multivariée intégrant plusieurs covariables. Les covariables de nature catégorielle ont été clairement identifiées. Dans les paramètres de prévision, nous avons sélectionné les probabilités ainsi que le groupe d'affectation. La prévalence combinée des immunoglobulines G et M constituait notre variable dépendante. L'analyse a mis en évidence plusieurs facteurs significativement associés à la séropositivité de la toxoplasmose, indépendamment du type d'anticorps considéré: L'âge (ORa : 1,10, IC 95% 1,03-1,18; p = 0,00); le contact avec les chats (Ora : 0,17, IC 95% 0,06-0,51; p = 0,00); le niveau de connaissances sur la toxoplasmose (ORa : 0,23, IC 95% 0,06-0,81; p = 0,02) et l'habitude de goûter la viande pendant sa cuisson (ORa: 0,45, IC 95% 0,22-0,90; p = 0,02) (Tableau 4).

**Tableau 4:** prédicteurs de la séroprévalence de *T. gondii* chez les femmes enceintes à Douala, Cameroun (N = 305)

Variables	OR brut (IC 95%)	Valeur p	ORa (IC 95%)	Valeur p
Age	0,64 (0,38-1,09)	0,10	1,10 (1,03-1,18)	0,00
Contact avec les chats	0,19 (0,09-0,41)	0,00	0,17 (0,06-0,51)	0,00
Niveaux de connaissances sur la toxoplasmose	0,39 (0,19-0,82)	0,01	0,23 (0,06-0,81)	0,02
Habitude de goûter la viande en cuisinant	0,58 (0,34-1,00)	0,05	0,45 (0,22-0,90)	0,02
Présence de chat à la maison	0,40 (0,23-0,72)	0,00	0,82 (0,33-2,01)	0,66
Consommation de fruits et légumes crus	0,68 (0,37-1,27)	0,23	0,55 (0,23-1,29)	0,17
Consommation de viande crue ou insuffisamment cuite	1,21 (0,64-2,29)	0,53	1,19 (0,51-2,79)	0,67
Consommation des repas dans la rue	0,94 (0,56-1,60)	0,84	0,72 (0,35-1,46)	0,37

OR: Odd ratio ORa: Odd ratio ajusté

## Discussion

Cette étude visait à déterminer la séroprévalence de l'infection à *T. gondii* et d'identifier ses facteurs associés chez les femmes consultant pour le suivi prénatal à l'Hôpital Gynéco-Obstétrique et Pédiatrique de Douala et à l'Hôpital Laquintinie de Douala. Notre recherche a examiné 305 femmes enceintes de 16 à 43 ans, avec un âge moyen de 29,45 ± 5,92 ans. Une recherche à Douala (Cameroun) [8] a révélé un âge moyen similaire de 29,9 ans, malgré un groupe différent (20-44 ans). Notre moyenne d'âge est inférieure à celle de Wam *et al*. [10] (31,13 ± 8,12 ans), mais dépasse celle d'Ayeah *et al*. [11] (28,05 ± 5,83 ans). Ces écarts résultent probablement des différentes tailles d'échantillon. Les grossesses variaient de 10 à 40 semaines d'aménorrhée, avec une moyenne de 25,78 ± 7,14 SA. La majorité des participantes (50,2%) traversait le deuxième trimestre, contrairement au Yémen [1] où 44,1% atteignaient le troisième trimestre. Cette différence s'explique par des périodes de conception distinctes. Pour l'éducation, 64,4% des femmes avaient suivi des études supérieures, contre 50% dans une étude comparable [9]. Cet écart reflète les inégalités d'accès aux universités selon les régions. Seulement 10% des participantes habitaient en zone rurale et peu étaient mariées, contrastant avec le Nord-ouest camerounais [10] où 94,9% vivaient en milieu rural et 53,9% étaient mariées. Ces contrastes s'expliquent par les spécificités géographiques et culturelles des régions étudiées. Notre étude montre que 50% des participantes n'avaient aucun antécédent obstétrical. Nous avons constaté un taux élevé d'avortements spontanés (31,5%) et de mortinaissances (3,3%), supérieur aux résultats de Nadia *et al*., [9] qui mentionnaient 11,6% et 2,5%. Leur étude a toutefois relevé un taux de malformations légèrement supérieur (0,8% contre 0,7% dans notre étude). Une autre recherche a identifié des taux encore plus importants d'avortements spontanés (32,5%) et de mortinaissances (5%) [11]. Ces différences s'expliquent possiblement par l'exposition variable à certains facteurs, notamment infectieux, selon les groupes étudiés. Concernant la parité, nos résultats indiquent une prédominance claire des multipares (55,7%) par rapport aux primipares (44,3%), ce qui confirme les données de Nadia *et al*. [9] et Ayeah *et al*. [11].

Cette similitude provient probablement de la forte proportion de multipares dans les échantillons analysés. Notre enquête révèle que 42,6% des participantes possédaient un chat domestique, et 33,1% interagissaient fréquemment avec ces animaux. Ces chiffres dépassent ceux d'une étude comparable [10], écart attribuable aux spécificités culturelles et aux habitudes de vie des populations étudiées. Par ailleurs, 38,4% Flatt *et al*. [5] (62% et 17,4%), différence probablement due aux caractéristiques propres aux zones d'étude. Notre recherche a également mis en évidence que 27,2% des participantes avaient bénéficié d'une transfusion sanguine, proportion supérieure à celle identifiée par Nadia *et al*. [9]. Les différences religieuses, l'accès inégal aux soins et les écarts de prix entre les lieux d'étude peuvent expliquer cette divergence. Quant à l'approvisionnement hydrique, les sources se caractérisaient par une diversité notable, l'eau de forage constituant la ressource principale pour 55,1% des cas, dépassant les 35,1% rapportés dans une étude similaire à l'ouest du Cameroun [9].

La séroprévalence d'anticorps anti-T gondii s'élevant à 74,4% (72,8% pour les IgG, 8,5% pour les IgM et 6,6% pour les IgG/IgM simultanément). Ce résultat demeure inférieur aux 78,6% identifiés par Nguefack *et al*. [12] à Douala, aux 80% documentés par Ayeah *et al*. [11] à Yaoundé, ainsi qu'aux 82,7% établis par Nadia *et al*., [9] à Dschang. Néanmoins, notre taux excède significativement ceux observés au Yémen (21,2%) [1] et dans une précédente étude camerounaise (54,3%) [10]. Ces disparités s'expliquent notamment par des variables géographiques, démographiques, comportementales et alimentaires propres aux populations étudiées. La diversité des méthodologies sérologiques employées contribue également à ces variations. Nous observons que 72,8% des parturientes présentent une immunité contre *Toxoplasma gondii* via les anticorps IgG. Cette proportion correspond aux 72,7% rapportés dans l'étude de Ayeah *et al*. [11], tout en surpassant les 62,8% identifiés par Nadia *et al*., [9]. Cette variation s'explique par la persistance caractéristique des anticorps IgG post-infection, leur présence signalant une contamination antérieure à la gestation. Quant aux IgM, notre prévalence de 8,5% surpasse les 1,3% relevés par Ayeah *et al*. [11], mais reste inférieure aux 11,6% d'une recherche comparable [9].

Notre étude révèle plusieurs facteurs associés à la séropositivité toxoplasmique. L'âge constitue un facteur significatif, confirmant les travaux de Ayeah *et al*. [11] qui l'identifient comme un risque statistique pour les IgG anti-*T. gondii*. Ces constats diffèrent des résultats de Nadia *et al*. [9] dans l'Ouest camerounais. Les femmes enceintes de 16 à 43 ans présentent un risque 1,107 fois supérieur de contracter la toxoplasmose. Ayeah *et al*. [11] rapportent des données similaires avec des rapports de cotes de 4,649 (15-24 ans) et 2,597 (25-34 ans). Le niveau de connaissance sur la maladie influence la séropositivité dans notre recherche, contrairement aux études de Dschang [9]. Le contact avec les chats domestiques augmente le risque d'infection, confirmant les résultats de Mustafa *et al*. [13] mais contredisant ceux de Wam *et al*. L'habitude de goûter la viande pendant la cuisson constitue également un facteur de risque, comme l'ont montré Nadia *et al*. [9]. Cette contamination provient des kystes de *T. gondii* présents dans les viandes crues ou mal cuites. Une proportion de 88,5% des participantes ignoraient la toxoplasmose, contre 11,5% qui la connaissaient bien. Ces chiffres dépassent ceux de Nadia *et al*. [9], où 57,9% des femmes manquaient d'information et 4,5% maîtrisaient le sujet. À Paris [14], 79,4% des futures mères comprenaient cette maladie. À Kinshasa [15], 84,9% des femmes n'avaient reçu aucune information sur ce pathogène, et seulement 5,2% le comprenaient en profondeur. Pour l'évaluation des connaissances sur la toxoplasmose, chaque participante donnait une seule réponse par question. Cette réponse peut différer des faits réels, particulièrement chez les femmes ayant déjà accouché, ce qui risque de créer un biais mémoriel.

## Conclusion

Notre étude a révélé une forte prévalence d'anticorps anti-*Toxoplasma gondii* au sein de la population étudiée. Plusieurs facteurs de risque ont été identifiés, notamment l'âge, l'interaction avec des félins, l'insuffisance de connaissances concernant la toxoplasmose, ainsi que la pratique consistant à goûter la viande en cours de cuisson chez les patientes suivies en consultation prénatale dans les établissements de santé concernés. Par ailleurs, nous avons constaté une méconnaissance générale de la toxoplasmose parmi les participantes. Il s'avère donc indispensable d'instaurer des programmes de prévention primaire afin de sensibiliser les femmes enceintes aux dangers potentiels de la toxoplasmose durant leur grossesse.

### Etat des connaissances sur le sujet

- L'âge, l'habitude de goûter la viande durant la cuisson et le contact avec les chats sont significativement associés à la séropositivité de la toxoplasmose;
- Qu'il soit estimé qu'environ un tiers de la population mondiale a été exposée à T. gondii;
- La toxoplasmose pendant la grossesse a été associée à une fausse couche, une hydrocéphalie, une calcification cérébrale et une choriorétinite chez le nouveau-né.

### Contribution de notre étude à la connaissance

- La prévalence globale des anticorps anti-T. gondii (IgG et/ou IgM) de 74,4%;
- Un faible niveau de connaissances sur la toxoplasmose;
- Le niveau de connaissances sur la toxoplasmose est associé à la prévalence de la toxoplasmose chez les femmes âgées de 16 à 43 ans.

## Remerciements

Nous tenons à remercier toutes les participantes de la présente étude.

## References

[1] Al-Adhroey AH, Al-Khaleq A, Mehrass O, Al-Shammakh AA, Ali AA, Akabat MYM (2019). Prévalence et prédicteurs de l'infection à Toxoplasma gondii chez les femmes enceintes de Dhamar, au Yémen. BMC Infect Dis.

[2] Con W, Dong X-Y, Meng W-F, Zhou N, Wang W Y, Huang S-Y (2015). Toxoplasma gondii, l'infection chez les femmes enceintes: une étude de séroprévalence et de cas-témoins dans l'est de la Chine. Biomed Res Int.

[3] Montoya JG, Liesenfeld O (2004). Toxoplasmose. The Lancet.

[4] Linguissi LS, Nagalo BM, Bisseye C, Kagoné TS, Sanou M, Tao I (2012). Seroprevalence of toxoplasmosis and rubella in pregnant women attending antenatal private clinic at Ouagadougou, Burkina Faso. Asian Pac J Trop Med.

[5] Flatt A, Shetty N (2013). Seroprevalence and risk factors for toxoplasmosis among antenatal women in London: a re-examination of risk in an ethnically diverse population. Eur J Public Health.

[6] Mwambe B, Mshana SE, Kidenya BR, Massinde AN, Mazigo HD, Michael D (2013). Sero-prevalence and factors associated with Toxoplasma gondii infection among pregnant women attending antenatal care in Mwanza. Tanzania, Parasites and vectors.

[7] Njunda AL, JCN Assob, DS Nsagha, Kamga HL, Nde PF, Yugah VC (2011). Séroprévalence de l'infection à Toxoplasma gondii chez les femmes enceintes au Cameroun. J Santé publique Afr.

[8] Liu Qn Wang Z-D, Huang S-Y, Zhu X-Q (2015). Diagnostic de la toxoplasmose et typage de Toxoplasma gondii. BMC.

[9] Nadia NAC, Nino LG, Cédric Y, Raoul SNS, Christian NO, Esther DD (2023). Séroprévalence des anticorps IgG et IgM de Toxoplasma gondii et facteurs de risque associés chez les femmes enceintes consultées dans trois centres de santé à Dschang. Cameroun, Parasite Epidemiol Control.

[10] Wam EC, Sama LF, Ali IM, Ebile WA, Aghangu LA, Tume CB (2016). Séroprévalence des anticorps IgG et IgM de Toxoplasma gondii et facteurs de risque associés chez les femmes en âge de procréer à Njinikom, dans le nord-ouest du Cameroun. BMC Res Notes.

[11] Ayeah JN, Oladokun A, Sumbele IUN, Ilesanmi AO, Bekindaka ON (2022). Séroprévalence de la toxoplasmose gestationnelle et néonatale et facteurs de risque à Yaoundé, Cameroun. J Parasitol Res.

[12] Nguefack CT, Meumeu IK, Ngaba GP, Kongnyuy E, Njamen TN (2016). Prévalence et facteurs associés à la vaccination contre Toxoplasma Gondii chez les femmes enceintes à Douala-Cameroun. J Womens Health Issues Care.

[13] Mustafa KM, Mohammed AB, Mero WMS (2024). Séroprévalence des anticorps anti-Toxoplasma gondii et facteurs de risque associés chez les femmes de la ville de Zakho, Irak. Cureus.

[14] Pouppel D (2012). Les connaissances des femmes enceintes sur la toxoplasmose en 2011. Paris: Ecole des Sages-Femmes.

[15] Badibanga KD, Botshudjama NP, Kamb TJ, Mutambel'Hity D, Kabamba MW (2025). Connaissances, Attitudes et Perceptions de la toxoplasmose chez les femmes enceintes et les personnels soignants à Kinshasa.

